# Lack of significant impact of deoxynivalenol (DON) on both swine dendritic cell activities and systemic infection caused by a virulent *Streptococcus suis* serotype 2 strain

**DOI:** 10.1186/s13567-026-01745-7

**Published:** 2026-04-07

**Authors:** Mélina Gilbert, Sonia Lacouture, Servan Payen, Younes Chorfi, Marcelo Gottschalk, Mariela Segura

**Affiliations:** 1 Research Group on Infectious Diseases in Production Animals (GREMIP) and Swine and Poultry Infectious Diseases Research Center (CRIPA), Department of Pathology and Microbiology, Faculty of Veterinary Medicine, University of Montreal, Saint-Hyacinthe, QC J2S 2M2 Canada; 2 Department of Biomedicine, Faculty of Veterinary Medicine, University of Montreal, Saint-Hyacinthe, QC J2S 2M2 Canada

**Keywords:** *Streptococcus suis*, deoxynivalenol (DON), dendritic cells, monocytes, cytokines, virulence

## Abstract

Deoxynivalenol (DON) is a common mycotoxin in swine feed known for its immunomodulatory effects and potential to exacerbate infectious diseases. However, its impact on systemic bacterial infections remains unclear. This study investigated whether DON influences the interaction between porcine marrow-derived dendritic cells (DCs) and *Streptococcus suis*, a major swine and zoonotic pathogen, as well as the progression of systemic infection in weaned piglets. In vitro exposure of DCs to non-toxic concentrations of DON (0.5 and 1 µM) neither altered the phagocytic uptake or intracellular survival of *S. suis*, nor did it affect anti-inflammatory cytokine expression. However, a moderate downregulation of IL-6 and IL-8 mRNA expression was observed. In vivo, piglets fed diets naturally contaminated with DON (1 or 2 mg/kg) and challenged with *S. suis* serotype 2 showed no significant differences in clinical signs, survival rates, or weight gain compared to controls. Although transiently increased bacteremia was observed at early stages post-infection in DON-exposed groups, it did not translate into more severe disease. These results suggest that under the conditions tested, DON has limited impact on DC functionality and does not significantly enhance susceptibility to systemic *S. suis* infection. This work provides relevant data for swine health risk assessments related to feed contamination with DON.

**Supplementary Information:**

The online version contains supplementary material available at 10.1186/s13567-026-01745-7.

## Introduction

Deoxynivalenol (DON) is a mycotoxin predominantly produced by the fungi *Fusarium graminearum* and *Fusarium culmorum* [1, 2]. It is recognized as one of the most common natural contaminants in wheat and other small grains cultivated in temperate regions [1]. At low levels, DON can cause anorexia, hinder weight gain, and stimulate the immune system [2]. Pigs are particularly vulnerable to DON due to its high oral absorption and species-specific differences in metabolizing this toxin [2]. When exposure levels rise to moderate or high (exceeding 0.84 mg/kg of feed), DON may result in reduced feed consumption or rejection, vomiting, and suppression of the immune system.

After oral ingestion of DON-contaminated feed, the gastrointestinal epithelial cell layer is the initial site of exposure [3]. Research indicates that DON can disrupt the intestinal barrier, impair immune function, and reduce both feed intake and weight gain [4]. Moreover, DON has been observed to alter gut microbiota diversity and shift the relative abundance of dominant microbial phyla [5]. Beyond its localized effects on the intestine, DON decreases the transepithelial electrical resistance of epithelial cells and, in a time- and dose-dependent manner, increases paracellular permeability, facilitating the translocation of pathogens such as pathogenic *Escherichia coli* across intestinal monolayers [6]. Additionally, in the presence of mycotoxins, the uptake of intracellular pathogens like *Salmonella* by macrophages is heightened, potentially leading to systemic infections, as these pathogens spread into the bloodstream [7].

The detrimental effects of DON and other mycotoxins are not confined to the intestinal phase. Indeed, the impact of this mycotoxin on immune cell functionality has also been documented. For instance, DON has been reported to disrupt the functions of porcine dendritic cells (DCs) both in vitro and in vivo, potentially contributing to its immunosuppressive effects [8]. Furthermore, DON can alter cytokine induction, chemotaxis, and the phagocytic activity of swine neutrophils [9].

Previous studies have demonstrated that oral administration of DON increases the susceptibility of pigs to infections caused by various viruses, including porcine reproductive and respiratory syndrome virus (PRRSv), porcine circovirus type 2 (PCV2), and porcine epidemic diarrhea virus (PEDv) [10–12]. The effect of DON on the severity of respiratory infections caused by *Mycoplasma hyopneumoniae* has also been investigated, although a clear synergic effect was not observed [13]. However, the impact of DON on infections caused by systemic bacterial pathogens in swine remains unexplored.

*Streptococcus suis* is one of the most important bacterial pathogens of swine, causing significant economic losses to the swine industry [14]. Moreover, it is a zoonotic agent representing serious risks for human health, especially in Southeast Asian countries [15]. *S. suis* causes various diseases in pigs, with meningitis, arthritis, and sudden death (septic shock) most frequently reported [14]. Among 29 described *S. suis* serotypes, serotype 2 is the most common type recovered from diseased pigs and humans [16]. Although the pathogenesis of *S. suis* has not been completely elucidated [17], the important role of cells of the innate immune system, such as DCs and neutrophils, during systemic infection caused by *S. suis* has been demonstrated [18, 19]. In addition, various predisposing factors may influence disease development, including environmental and management conditions as well as co-infections [14, 20]. Since mycotoxins have recently been hypothesized to play a role in *S. suis* intestinal infections [21], the aim of this study was to evaluate whether DON can modulate the interactions between *S. suis* and swine DCs. Additionally, the effect of in-feed administration of moderate and high levels of DON on the clinical course of an experimental *S. suis* systemic infection in weaned piglets was evaluated.

## Materials and methods

### Bacterial strains and growth conditions

Two strains of *S. suis* serotype 2 were used in this study: strain P1/7, which is a virulent and well characterized strain [22] and, for comparison purposes and technical controls for certain in vitro experiments, its non-encapsulated isogenic mutant strain, ∆*cpsF* [23]. *S*. *suis* strains were cultured in Todd Hewitt broth (THB; Becton Dickinson, Mississauga, ON). For in vitro cell culture assays, bacteria were prepared as previously described [23] and resuspended in cell culture medium. For experimental infections, early stationary phase bacteria were washed twice in phosphate-buffered saline (PBS) pH 7.4 and resuspended in THB, as previously described [24]. Bacterial cultures were appropriately diluted and plated on Todd-Hewitt agar (THA) to accurately determine bacterial concentrations.

### Bone marrow-derived dendritic cells (DCs)

To produce bone marrow-derived DCs, piglets between 5 and 9 weeks of age which originated from a high health status herd free of PRRSv and without endemic clinical infections caused by *S. suis* serotype 2 were used. Manipulation of animals were done in accordance with the recommendations of the guidelines and policies of the Canadian Council on Animal Care and the principles outlined in the Guide for the Care and Use of Laboratory Animals (see below).

The extraction and culture of DCs from the bone marrow of pig femurs was performed as described [25]. Briefly, after removal of muscles, femurs were sliced into pieces and shaken in 1 L of PBS for 2 h at room temperature. The suspension containing the bone marrow stem cells was recovered by filtration using sterile gas, and the cells were then centrifuged at 250 × *g* for 10 min at 4 °C. Red blood cell lysis was performed with a kit (eBioScience, San Diego, CA, USA), then cells were washed, filtered on a 40 µm cell strainer (Falcon, Mississauga, ON, Canada), suspended at approximately 2 × 10^7^ cells/mL in cryopreservation solution containing 95% fetal bovine serum (FBS, Gibco Burlington, ON, Canada) and 5% dimethyl sulfoxide (DMSO, Sigma-Aldrich, St. Louis, MO, USA) and stored in liquid nitrogen. For the experiments, bone-marrow-derived stem cells were thawed and centrifuged at 250 × *g* for 10 min at 4 °C. Cells were suspended in a complete medium with antibiotics containing RPMI 1640 (Gibco), 10% FBS (v/v), 10 mM HEPES, 2 mM L-glutamine (Gibco), 0.001 mg/mL gentamicin and 100 U/mL penicillin–streptomycin at a concentration of 1 × 10^6^ cells/mL in a 6-well plate (Falcon). Porcine Granulocyte–Macrophage Colony Stimulating Factor (GM-CSF), produced in our laboratory from the CHO-K1/pGM-CSF cell line as previously described [26], was added at a 1:50 dilution to each well, and the cells were incubated at 37 °C under 5% CO_2_. On days 3 and 6, the culture medium was replaced with complete RPMI medium fresh with antibiotics to remove non-adherent cells. On day 8, immature DCs were harvested via up and down pipetting for future experiments. The phenotype of DCs was confirmed by FACS as CMH-I^+^, CMH-II^+^, SWC3^+^, CD1^+^, CD16^+^, CD14^+^, CD11R1^−^ and CD4a ^low/−^, as previously described [25]. The methodology used does not exclude the presence of certain contaminants of other cells, such as macrophages, but the culture is predominantly enriched in immature DCs. Immature DCs were adjusted to a concentration of 1 × 10^6^ cells/well in a total volume of 500 µL in a 24-well plate (Falcon).

### Preparation of the DON stock solution for in vitro use

For in vitro experiments, DON was purchased from Sigma-Aldrich. A 10 mM stock solution of DON was prepared in DMSO. It was then portioned into 50 µL aliquots and stored at -20 °C. For the experiments, serial dilutions of the DON stock solution were prepared in the corresponding cell culture medium to allow the addition of a similar volume of vehicle to each culture well.

### DON treatment of cells

The experimental conditions for DON pretreatment were previously established based on cell activation (see below) and cytotoxicity, by measuring the release of the cytosolic enzyme lactate dehydrogenase (LDH) [25]. All the results presented were obtained under non-toxic conditions. For phagocytosis, intracellular survival studies and cytokine induction, and before adding mycotoxin, porcine DCs were washed once with sterile PBS preheated to 37 °C (1 mL/well). Then, 1 mL of complete RPMI medium preheated to 37 °C, without antibiotics, containing DON at a concentration of 0.5 µM or 1 µM (maximal DON concentration that did not induce high cytotoxic effects under the conditions used), was added to the culture wells for an incubation period of 24 h, at 37 °C under 5% CO_2_.

### Phagocytosis and intracellular survival tests

Phagocytosis and intracellular survival assays were performed as described previously [27]. Briefly, porcine DCs underwent DON treatment (or not) as described in the previous section. *S. suis* P1/7 and its non-encapsulated ∆*cpsF* mutant were adjusted to a final concentration of 1 × 10^7^ CFU/mL, corresponding to a MOI 10. Bacterial concentrations were chosen based on previously published results in our laboratory [27] and preliminary data obtained during standardization of the test. Bacteria were then pre-opsonized with 20% fresh whole serum from 5-week-old piglets in culture medium. To confirm that the serum used was negative for *S. suis* specific antibodies (either maternal-derived or actively produced), an ELISA specific to the strains used was previously carried out to confirm the very low total immunoglobulin titers (< 1/200) as previously described [28]. Opsonization was carried out for 30 min at 37 °C, then the bacterial suspension was added to the wells (1 mL/well) and the plates were centrifuged for 10 min with shaking at room temperature to allow bacteria to come into contact with the cells adhered to the bottom of the wells [25]. The infected 24-well cell culture plates were centrifuged and incubated for 60 min at 37 °C under 5% CO_2_ (optimal time for *S. suis* phagocytosis determined by previous phagocytosis assays). Cells were then washed twice with PBS and culture medium with antibiotics [gentamicin (100 µg/mL) and penicillin G (5 µg/mL)] was added to the cells. The cells were incubated for 2 h at 37 °C under 5% CO_2_. After antibiotic treatment, cells were washed three times with PBS, and the last wash was plated onto THA to confirm the effectiveness of the antibiotic treatment. The cells were then scraped and the CFU count of the bacteria was determined in the same way as described above. The phagocytosis rate of *S. suis* was expressed as CFU/mL.

For intracellular survival assays, a phagocytosis test was first performed as described above. After 60 min contact between cells and bacteria, the antibiotics were added, except that the treatment was extended for different incubation times up to a maximum of 7 h. The cells were then treated as described above and viable counts were performed to monitor changes in the viability of bacteria inside the immune cells. The results come from at least four independent experiments [27].

### Induction of pro-inflammatory cytokines

For cell activation, porcine DCs were either mock-infected, treated with DON, infected with *S. suis* or both (treated with DON and infected with *S. suis*). To do this, cells were first treated with DON, where appropriate, as described above. The culture medium was then removed from the wells to be replaced with 1 mL of either cell culture medium or a bacterial suspension of *S. suis* P1/7 at an MOI 1. The 24-well plates were then centrifuged for 10 min at room temperature and incubated for 6 h at 37 °C under 5% CO_2_ to allow the induction of messenger RNA (mRNA). The bacterial concentration and incubation time were chosen based on previously published results from the laboratory [27, 29] and results obtained during standardization of cell culture conditions. After incubation, cells were washed once with PBS, and RNA from the cells was extracted using a silica column, according to the manufacturer’s instructions (Aurum total RNA mini kit, Bio-Rad, Mississauga, ON, Canada). Briefly, cells were lysed with a solution containing β-mercaptoethanol and 70% ethanol. The cell lysate was transferred to a silica column and washed with low- and high-stringency wash solutions from the kit. DNase solution was added to the cell lysate to retain only the RNA. RNA was eluted into 40 µL of elution solution, quantified using the NanoDrop 1000 (Fisher, Ottawa, ON, Canada), diluted in RNase-free water to 100 ng/µL and stored at − 80 °C. Complementary DNA (cDNA) was synthesized from 200 ng of RNA with the M-MLV reverse transcriptase kit (Invitrogen, Carlsbad, CA, USA) using Oligo (dT)12–18 Primer 25 µg (0.5 µg/µL) and dNTP Mix, 10 mM each (Thermoscientific, Vilnius, Lithuania), according to the manufacturer’s instructions. Endotoxin-free reagents were used for cell culture experiments.

The primers used for the q-PCR analysis are listed in Table 1. The qPCR was performed as described, with some modifications [29]. Briefly, the CFX96 thermal cycler (Bio-Rad Laboratories, Hercules, CA, USA) showed that the primers had an efficiency between 90 and 110%. The cDNA was amplified using the PowerTrack SYBR Green Master Mix Kit (Thermofisher). The cDNA amplification program was performed as follows: a 2 min enzyme activation step at 95 °C, followed by 40 cycles of 15 s denaturation at 95 °C and a 1 min annealing/extension step at 58 °C. The two genes β2M and PPiA were used as normalization genes to compensate for potential differences in the cDNA amounts between the different samples. Differences in gene expression were calculated using the normalized gene expression calculation method (∆∆Cq) of CFX Maestro software (v.2.1: Bio-Rad). Mock infected cells were used as negative control and calibrator for the analysis. Results represent at least four independent experiments.

**Table 1:** **List of primers used in this study to evaluate expression of mRNA by RT-PCR**

Gene Name	Forward	Reverse
*PPiA*	TGC AGA CAA AGT TCC AAA GAC AG	GCC ACC AGT GCC ATT ATG G
*β2M*	CGT GGC CTT GGT CCT GCT CG	TCC GTT TTC CGC TGG GTG GC
*IL-6*	ACT CCC TCT CCA CAA GCG CCT T	TGG CAT CTT CTT CCA GGC GTC CC
*CXCL8-8*	TGT GAG GCT GCA GTT CTG GCA AG	GGG TGG AAA GGT GTG GAA TGC GT
*IL-10*	GCT GCG GCG CTG TCA TCA ATT	ACC CAT GGC TTT GTA GAC ACC CC

### Animals used for experimental infection

This study was carried out in accordance with the recommendations of the guidelines and policies of the Canadian Council on Animal Care and the principles outlined in the Guide for the Care and Use of Laboratory Animals. The protocols and procedures were approved by the Animal Welfare Committee of the University of Montreal (protocol number Rech-1570). Recently weaned, three-week-old, Landrace/white mixed breed piglets of either sex were acquired from a high health commercial farm in Quebec, with no history of endemic clinical problems caused by *S. suis*, no vaccination program against this pathogen and free of PRRSv. Animals from this source were previously successfully used for *S. suis* infections [24].

### Experimental diets

Experimental diets (Table 2) were formulated according to the energy and amino acid requirements for piglets as previously described in the National Swine Nutrition Guide [30]. Wheat and corn used in experimental diets were naturally contaminated with DON. Dietary contents of DON and other mycotoxins were analyzed by MasterLab (St-Hyacinthe, Qc, Canada) and presented in Table 3.

**Table 2:** **Diet compositions**

Ingredients (Kg/Kg of diet)	Low DON diet	Intermediate DON diet	High DON diet
Corn (< 0.5 ppm DON)	0.20	0.03	0.00
Corn (5.8 ppm DON)	0.00	0.17	0.20
Wheat (< 0.5 ppm DON)	0.20	0.20	0.00
Wheat (9.0 ppm DON)	0.00	0.00	0.20
Piglet PREMIX ^1^	0.60	0.60	0.60

^1^ Piglet premix contains crude protein (29.50%); fat (2.45%); crude fiber (1.95%); calcium (1.29%); phosphorus (0.87%); sodium (0.57%); Zinc (833 mg/kg); Iron (387 mg/kg); Manganese (74 mg/kg); copper (199 mg/kg); Iodine (1.0 mg/kg); Cobalt (0.6 mg/kg); vitamin A (16,766 U.I./kg); vitamin D (2500 U.I./kg); vitamin E (200 U.I./kg).

**Table 3:** **Mycotoxin contents of the diets used in the current study**

	Mycotoxin concentrations in ppm in diet		
Mycotoxins	Low DON	Intermediate DON	High DON
**Deoxynivalenol (DON)**	**0.197**	**1.06**	**2.08**
Acetyl-Deoxynivalenol	0.0911	0.117	0.191
Deoxynivalenol-3-glucoside	< 0.06	0.143	0.223
Aflatoxin B1	< 0.0010	< 0.0010	< 0.0010
Aflatoxin B2	< 0.0025	< 0.0025	< 0.0025
Aflatoxin G1	< 0.0025	< 0.0025	< 0.0025
Aflatoxin G2	< 0.0030	< 0.0030	< 0.0030
Fumonisin B1	0.0676	0.0688	0.0437
Fumonisin B2	< 0.051	< 0.051	< 0.051
Ochratoxin A	< 0.0040	< 0.0040	< 0.0040
T-2	0.00660	0.00691	0.00698
HT-2	< 0.010	0.01090	0.01140
Zearalenone	0.029	0.187	0.384
Diacetoxyscirpenol	< 0.0050	< 0.0050	< 0.0050
Sterigmatocystin	< 0.0050	< 0.0050	< 0.0050
Mycophenolic Acid	< 0.0300	< 0.0300	< 0.0300

### Experimental infection

Upon arrival, piglets were individually weighed, tagged, and assigned to three groups (*n* = 36; 12 animals/group) with equal average weight (approximately 6 ± 0.5 kg) and proportional distribution of males and females. After two weeks of acclimation (Day 0), blood samples were taken and piglets were weighed again and fed ad libitum with naturally contaminated diets containing less than 0.5 (control diet), 1 or 2 mg/kg of DON (which correspond to < 1, 1 or 2 ppm, respectively) for the duration of the experiment. During the following weeks, blood samples for DON measurement were taken (13 days after the beginning of the contaminated diet and before challenge) and animals were weighted, weekly. After two weeks on the experimental diets, all groups were inoculated intramuscularly (IM) with 1 ml containing 1 × 10^8^ CFU of a log-phase culture of *S. suis* serotype 2 strain P1/7. For bacteremia studies, blood samples were taken at 24 h and 48 h post-infection and plated as previously described [24]. Pigs were monitored three times per day over a period of five days for the presence of clinical signs and mortality. A daily clinical score was calculated based on a clinical observation sheet [24]. Assessed signs were general behavior, locomotion (musculoskeletal alterations) and functional alteration of the central nervous system (CNS). Behavior clinical scores were given as follows: 0 = normal attitude and response to stimuli; 1 = slight depression with marginally delay in the response to the stimuli but preserved appetite; 2 = moderate depression, animal only responds to repeated stimuli, reluctant to move, decreased appetite; 3 = severe depression, non-responsive, recumbent, diminished appetite. Locomotion clinical scores were given as follows: 0 = normal gait and posture; 1 = one joint affected, light lameness, but rises and moves without assistance; 2 = moderate lameness, 2–3 joints affected with the swelling but stands without assistance; 3 = severe lameness, ataxia 3–4 joints affected, recumbent and cannot stand or move. Finally, CNS clinical scores were given as follows: 0 = normal physiological behavior and response to stimuli; 1 = slight incoordination, strabismus; 2 = moderate incoordination, trembling; 3 = severe, lateral or dorsal head inclination, ataxia, opisthotonus, nystagmus, convulsions [24]. Pigs having a clinical score = 3 in locomotion and general behavior for two consecutive days or a CNS clinical score = 3 were humanely euthanized. Euthanized and dead animals were given a score of 10. Ketamine (30 mg/kg; Narketan®, Vetoquinol, Lavaltrie, QC, Canada) and xylazine (5 mg/kg; Rompun®, Bayer, Mississauga ON, Canada) were administered IM to achieve complete anesthesia followed by intracardiac administration of pentobarbital sodium (60 mg/kg; Dorminal, Rafter 8 product, Calgary AB, Canada).

### DON and Deepoxy-deoxynivalenol (DOM-1) measurements

Blood samples taken after acclimation and two weeks of DON treatments were transported on ice to the laboratory where serum was separated by centrifugation (2000 × *g* for 10 min) and stored at − 80 °C for further analyses. For DON and DOM-1 measurements, 1 mL of each serum sample was mixed with β-glucuronidase enzyme and incubated overnight at 37 °C. DON and DOM-1 were extracted by immunoaffinity columns (Viacam, Sacramento, CA, USA), and samples were evaporated and re-suspended in a mobile phase buffer containing acetonitrile and water (13:87). They were then analyzed by high-performance liquid chromatography at 218 nm using a variable wavelength detector [31].

### Statistical analysis

All in vitro data are expressed as mean ± SEM. Data from phagocytosis, intracellular survival, and cytokine gene expression were analyzed by an unpaired non-parametric Mann–Whitney test. Average values represent the result of at least three independent experiments. Data for RT-qPCRs were subjected to ANOVA procedures. For in vivo animal experiments, survival rates were evaluated with chi-square analysis using the Kaplan–Meier method, and the significance of the difference was tested using the log-rank test. The clinical scores were transferred by ranking, and the significance of the difference between groups was determined by a non-parametric Kruskal–Wallis test with Dunn’s multiple comparisons using GraphPad Prism 8 software (GraphPad Software, San Diego, CA, USA). Data were considered significantly different if *p* < 0.05.

## Results

### Effect of DON on phagocytosis and intracellular survival of *S. suis* by DCs

**Figure 1:**
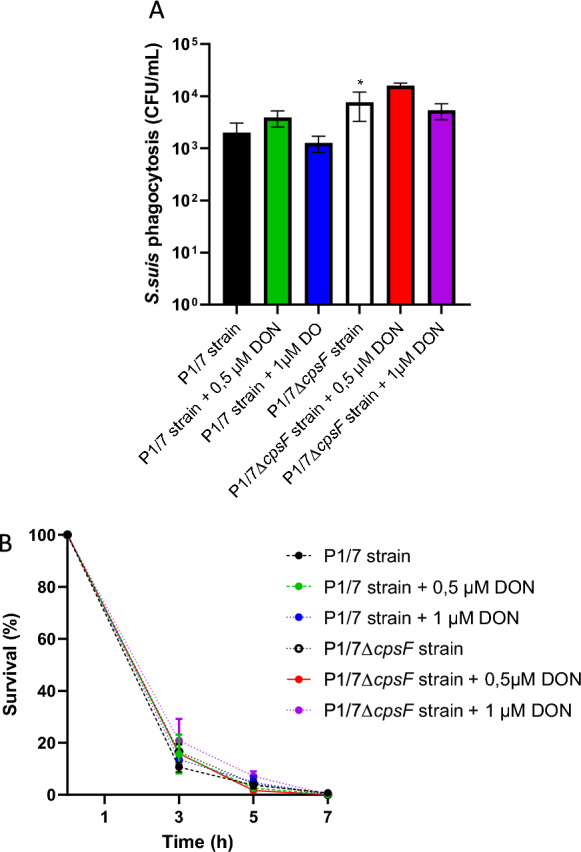
**Effect of DON on dendritic cell phagocytosis and intracellular survival of** ***S. suis.*** **A**Phagocytosis of *S. suis* P1/7 wild-type (WT) strain and its Δ*cpsF* non-encapsulated mutant by DCs. After treatment (or not) of DCs with DON for 24 h, cells were infected with *S. suis* for 60 min at an MOI of 10. The phagocytosis rate of *S. suis* in the presence of fresh porcine serum is expressed as colony-forming units (CFU/mL); **B** Intracellular survival of *S. suis* P1/7 strain and its Δ*cpsF* non-encapsulated mutant within DCs. After treatment (or not) of DCs with DON for 24 h, cells were infected with *S. suis* for 60 min at an MOI of 10 in the presence of fresh porcine serum. Antibiotics were then added for 3 h, 5 h and 7 h. The survival rate of 100% represents the phagocytosis rate (similar to that observed in the previous experiment, Figure 1A) after 2 h of antibiotic treatment. For both experiments, data represent the mean ± SEM (*n* = 4). Other than a significant higher level of phagocytosis with the Δ*cpsF* non-encapsulated mutant when compared to the P1/7 strain (*p < 0.05), no other significant differences were observed with presence or absence of DON using non-parametric Mann-Whitney test.

Phagocytosis rate of *S. suis* by DCs was evaluated (Figure 1A). DON had no influence on the phagocytosis rate of the P1/7 strain at any concentration. Similarly, the non-encapsulated mutant was, as expected, phagocytosed at significantly higher levels than the wild-type strain, but DON did not affect the general level of phagocytosis (Figure 1A).

Resistance to the intracellular killing mechanisms of phagocytes may contribute to the pathogenesis of the infection. Therefore, the intracellular survival rate of *S. suis* in the presence or absence of DON treatment was assessed in DCs. To compare the intracellular survival rate of *S. suis* P1/7 or *∆cps2F*, a value of 100% was assigned to the phagocytosis rate observed after 60 min of incubation. As previously reported [32], intracellular survival after phagocytosis in the absence of DON (control group) was not significantly different for both (wild-type and non-encapsulated mutant). Similar results were obtained for both strains when cells were pre-treated with DON, indicating that the presence of the mycotoxin does not influence intracellular killing (Figure 1B).

### Effect of DON on modulation of the relative mRNA expression of cytokines induced by *S. suis*

**Figure 2:**
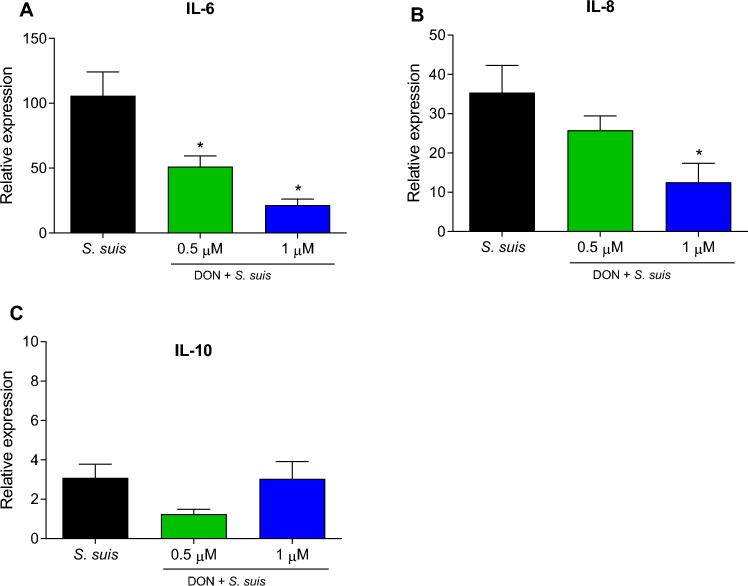
**Effect of DON on cytokine gene expression in dendritic cells infected with** ***S. suis.*** Relative expression of IL-6 (**A**), IL-8 (**B**) and IL-10 (**C**) cytokines by DCs as measured by RT-qPCR. After treatment of DCs with DON for 24 h, cells were infected (or not) with *S. suis* P1/7 strain for 6 h at an MOI of 1. Data represent mean ± SEM (*n* = 3, with 3 technical replicates each). *p < 0.05 shows a significant difference between cells infected with *S. suis* only and cells pre-treated with DON and then infected with *S. suis* using non-parametric Mann–Whitney test.

The relative mRNA expression of two pro-inflammatory cytokines (IL-6 and IL-8) as well as one anti-inflammatory cytokine (IL-10) by DCs activated with *S. suis* in the presence or absence of DON was measured. The mRNA expression of IL-6 significantly decreased in the presence of 0.5 and 1 µM of DON, whereas that of IL-8 decreased at a concentration of 1 µM of DON. On the other hand, the expression of IL-10 was low and remained unchanged (Figure 2).

### Confirmation of the concentration of DON in different feed preparations, blood levels of DON and DOM-1 and daily weight gain in animals

**Figure 3:**
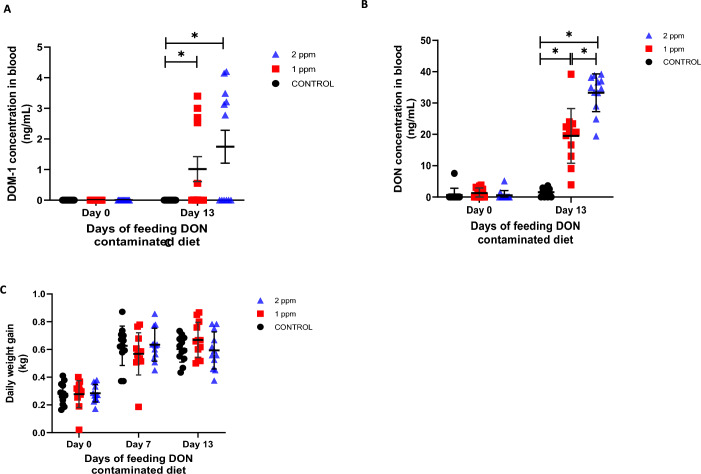
**Serum DON and DOM-1 levels and growth performance in pigs fed DON-contaminated diets.** **A** DON and **B** DOM-1 measurements; each serum sample was mixed with β-glucuronidase enzyme and incubated overnight at 37 °C. DON and DOM-1 were extracted by immunoaffinity, and samples were evaporated and re-suspended in a mobile phase buffer containing acetonitrile and water (13:87). They were then analyzed by high-performance liquid chromatography at 218 nm using a variable wavelength detector. *p < 0.05 showed significant differences between groups. **C** Daily weight gain of control animals and those fed with 1 ppm or 2 ppm of DON-contaminated diet.

DON concentrations in feed preparations were confirmed to be those expected (Table 3). In addition, no significant concentrations of other mycotoxins were observed (Table 3). DON levels in animal blood samples corresponded to feed contamination levels with DON: low (control), intermediate (1 ppm of DON) or high (2 ppm of DON) levels were detected (Figure 3A). DOM-1 is a detoxification metabolite of DON which also shows the exposure to DON and the extent of which this mycotoxin is metabolized [33]. Both groups treated with DON presented significant higher levels of this metabolite than the control group (*p* < 0.05). No statistical differences were observed between the two DON treated groups (Figure 3B). Finally, no differences in daily weight gain were observed among the groups (Figure 3C).

### Effect of DON on animal susceptibility to systemic infection caused by *S. suis* serotype 2

The goal of our study was to evaluate if feeding animals with DON contaminated diets increases the susceptibility of pigs to a systemic infection with *S. suis* serotype 2. The intramuscular challenge model used in this study was able to reproduce typical clinical signs and lesions caused by *S. suis* infection in weaned piglets as those observed in the field [14]. Unexpectedly, similar mortality results than those previously obtained when a significant higher dose and the intraperitoneal route of infection were used [24]. Necropsy of affected and euthanized animals revealed typical polyarthritis lesions with abundant fibrinopurulent exudate in joint cavities; spleen was enlarged, with petechial hemorrhages indicating systemic infection (septicemia), similarly to what was previously observed [24].

**Figure 4:**
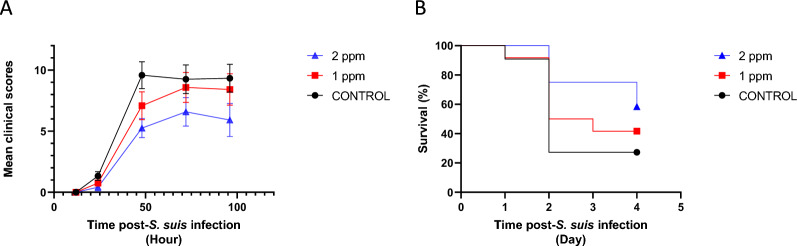
**DON does not increase susceptibility to systemic infection caused by** ***S. suis*** **serotype 2**. **A** Mean clinical scores and **B** survival rate of control animals and those fed during two weeks with 1 ppm or 2 ppm of DON-contaminated diet after an intramuscularly experimental infection with 1 ml containing 1 × 10^8^ CFU of a log-phase culture of *S. suis* serotype 2 strain P1/7. Pigs were monitored three times per day over a period of five days for the presence of clinical signs and mortality. A daily clinical score was calculated based on a clinical observation sheet [24], as described in material and methods. No significant differences were observed between the groups (*p* > 0.05). Mortality (long-rank (Mantel-Cox) was not significantly different between animals fed with 1 or 2 ppm of DON and it was not statistically higher than the control group (*p* > 0.05).

Twenty-four and 48 h after infection, bacteremia levels were statistically similar in all three groups (Additional file 1) (*p* > 0.05). When the presence of clinical signs was evaluated, most challenged animals from all groups showed signs of depression, incoordination, and shifting lameness. In more severe cases, there were signs of septicemia and meningitis, characterized by convulsion, head inclination, ataxia, opisthotonos, paddling, and nystagmus. Indeed, mean clinical scores of animals fed with DON-contaminated feed did not show major differences when compared to the control group (Figure 4A) (*p* > 0.05). The lack of effects on clinical signs was also observed in mortality rates, which was not significantly different between animals fed with 1 or 2 ppm of DON and it was not statistically higher than the control group (*p* > 0.05), as evaluated by the long-rank (Mantel-Cox) test (Figure 4B).

## Discussion

*S. suis* is a major bacterial pathogen in post-weaned piglets, responsible for significant economic losses in the swine industry worldwide [14]. *S. suis* disease alone accounts for about 1/3^rd^ of the total antibiotic usage in weaner pigs [34]. While the bacterium is widespread and can be found in the upper respiratory tract of healthy pigs, not all strains present high virulence potential [14]. Indeed, some strains are considered as opportunistic and clinical disease does not always occur upon exposure [35]. This suggests that additional factors also contribute to disease development. Indeed, various predisposing conditions—such as environmental stress, poor management practices, and co-infections with viruses or other pathogens—have been implicated in triggering or exacerbating *S. suis* infections [20]. These factors can compromise the immune system or disrupt mucosal barriers, facilitating bacterial invasion and progression to systemic disease. Mycotoxins, particularly DON, are secondary fungal metabolites that commonly contaminate cereal grains used in swine feed. Among livestock species, pigs are notably sensitive to DON due to their efficient gastrointestinal absorption and comparatively low detoxification capacity. Nursery pigs are most highly susceptible to both *S. suis* infection and DON contamination in feed [36].

DON has previously been associated with immunomodulatory and immunosuppressive effects in pigs, especially in the context of viral infections and gut-associated bacterial pathogens, [11, 37]. However, a two-way immunotoxicity has also been reported, defined as mycotoxins not only inhibiting immunity but also stimulating immunity and inducing inflammation [38]. This study investigated, for the first time, the potential impact of DON on the interaction between DCs and a systemic bacterial pathogen, *S. suis*, as well as on the progression of systemic infection in vivo. In vitro, exposure of porcine DCs to non-toxic concentrations of DON (0.5 and 1 µM) did not influence the phagocytic capacity of *S. suis*. Higher concentrations of DON resulted in high cytotoxicity levels which prevented any further studies with cells (unpublished results). Although well-encapsulated *S. suis* is not easily phagocytosed by immune cells [25], results from the current study could not demonstrate any significant reduction in the phagocytosis capacity of DCs, as shown with a non-encapsulated mutant of *S. suis*, which has been previously described as easily ingested by immune cells [25]. These results differ from previous findings suggesting that DON can impair phagocytic activity of other immune cells, such as alveolar macrophages and neutrophils [9, 39]. However, our study is the only one carried out so far with DON contaminated immune cells and live bacteria, which may highly influence the results. Finally, intracellular killing of phagocytosed *S. suis* was not affected by the presence of DON, indicating that the bactericidal activity of DCs was not altered by the presence of this mycotoxin. This is the first study on the effect of DON on the capacity of an immune cell to kill phagocytosed bacteria.

In addition, a slight immunomodulatory effect of DON was observed, mainly regarding the expression of IL-6 and, to a lesser extent, IL-8. Although the protein expression has not been evaluated, these small differences in mRNA expression are usually not detected by ELISA due mainly to the low sensitivity of these immunoassays [40]. Other studies reported that DON triggers rather than inhibits the expression of pro-inflammatory cytokines [41–43]. However, it should be taken into consideration that this mycotoxin plays immunostimulatory and immunosuppressive roles at different exposure doses. For example, low doses of DON exposure enhance TNF-α and IL-6 expressions in vivo and in vitro and increase the chemotaxis and phagocytosis of pig alveolar macrophages while promoting macrophage polarization to M1. However, high doses of DON exposure enhance transforming growth factor beta and IL-10 (anti-inflammatory cytokines) expressions in vivo (3000 μg/kg diet) and in vitro, suppress the chemotaxis and phagocytosis of pig alveolar macrophages, and promote macrophage polarization to M2 [10, 38]. It should be noted that, in the current study, the presence of DON did not influence the expression of IL-10. Overall, cytokine transcriptional data should be interpreted with caution in the absence of measurable functional outcomes. Moreover, immunological changes observed in vitro in closed experimental systems may not always translate into detectable effects in vivo, due to the complexity and redundancy of host immune responses.

In vivo, piglets exposed to diets naturally contaminated with DON at concentrations of approximately 1 ppm and 2 ppm in feed showed no significant differences in clinical scores, survival rates, or daily weight gain compared to the control group following experimental systemic infection with *S. suis* serotype 2. Accordingly, levels of bacteremia during the first 48 h post-infection were not significantly different. This may reflect a relatively low impact of DON on the first reaction of host innate defenses. These results agree with those obtained in animals infected with *M. hyopneumoniae* and PCV2 [13, 44]. Interestingly, feed contaminated with DON increases the effect of PRRSv infection on weight gain, lung lesions and mortality, without increasing significantly viral replication [12]. It is important to mention that this effect on PRRSv infection may influence, in the field, the susceptibility of animals to *S. suis* [20]. Animals used in the current study were PRRSv free.

It is not clear why the obtained effects of dietary DON vary among previously published experiments with different pathogens. It might be explained by different factors such as starting weight or age of the pigs, the contamination source of DON (natural versus artificial contamination), presence of other known or unknown undetected fungal metabolites or pathogens, duration of the study (adaptation), number of pigs used in the study, gender of the pigs, health status, nutritional balance of the pig and statistical design of the study [3]. In addition, it is also possible that the virulence of the *S. suis* P1/7 strain used in this study was sufficiently high to mask subtle immunomodulatory effects induced by DON. Indeed, the model used in the present study was aimed to obtain a relatively lower mortality rate to clearly observe a possible impact of DON contamination in feed, by using a lower infectious dose (a reduction of > 1.5 log of bacteria) and the intramuscular (instead of intraperitoneal) route of infection, as it was previously reported by our laboratory [24]. Surprisingly, animals were still seriously affected, and the mortality rate was similar to that previously reported [24]. Future studies could explore the use of more opportunistic (with lower virulence potential) strains, such as North American ST25 or ST28 serotype 2 strains [45], in combination with other stressors (e.g., co-infections, poor nutrition, or additional mycotoxins), that might synergize with DON to increase the susceptibility to systemic bacterial streptococcal infections. However, conditions to standardize experimental infections in pigs using conventional pigs with these types of strains have not been published so far.

In addition, the predisposing role of DON (and other mycotoxins) may be local at the intestinal mucosa, as recently proposed [21], rather than systemic. Indeed, it has already been reported that DON promotes translocation of gut pathogens such as *E. coli* and *Salmonella* in intestinal models [6, 46]. Finally, the presence of more than one mycotoxin, as it is usually observed in the field [47, 48], can induce a synergistic effect that could not be studied in the present report. Indeed, a recent study on the in vitro effect of DON on the interaction of *S. suis* with epithelial cells, showed that two mycotoxins (DON and T-2) must be present to observe an effect on the transepithelial electrical resistance and bacterial translocation [21]. Further studies using a mucosal infection model, such as the oral route, are warranted to evaluate the effect of DON (and other mycotoxins) on the pathogenesis of intestinal infection caused by this pathogen.

In conclusion, our findings suggest that DON, at levels commonly found in swine feed and under the precise conditions use in the current study, has limited impact on the interaction of *S. suis* with porcine DCs and does not significantly influence the course of systemic infection under experimental conditions using a high virulent strain. Further in vivo studies using less virulent strains and/or an oral infection model are warranted.

## Supplementary Information

**Additional file 1 Effect of DON-contaminated feed on blood bacterialburden afterS. suisinfection**.

## Data Availability

No datasets were generated or analysed during the current study.

## Abbreviations

DON: deoxynivalenol
DOM-1: deepoxy-deoxynivalenol
PRRSv: porcine reproductive and respiratory syndrome virus
PCV2: porcine circovirus type 2
THB: Todd Hewitt broth
THA: Todd Hewitt agar
DC: dendritic cells
GM-CSF: porcine granulocyte–Macrophage colony stimulating factor
ppm: part per million
LDH: enzyme lactate dehydrogenase
IL: interleukin
CFU: colony-forming units

## References

[1] Kostelanska M, Dzuman Z, Malachova A, Capouchova I, Prokinova E, Skerikova A, Hajslova J (2011) Effects of milling and baking technologies on levels of deoxynivalenol and its masked form deoxynivalenol-3-glucoside. J Agric Food Chem 59:9303–9312 21797213 10.1021/jf202428f

[2] Wang P, Yao Q, Meng X, Yang X, Wang X, Lu Q, Liu A (2023) Effective protective agents against organ toxicity of deoxynivalenol and their detoxification mechanisms: a review. Food Chem Toxicol 182:114121 37890761 10.1016/j.fct.2023.114121

[3] Antonissen G, Martel A, Pasmans F, Ducatelle R, Verbrugghe E, Vandenbroucke V, Li S, Haesebrouck F, Van Immerseel F, Croubels S (2014) The impact of *Fusarium* mycotoxins on human and animal host susceptibility to infectious diseases. Toxins (Basel) 6:430–452 24476707 10.3390/toxins6020430 PMC3942744

[4] Holanda DM, Kim SW (2021) Mycotoxin occurrence, toxicity, and detoxifying agents in pig production with an emphasis on deoxynivalenol. Toxins (Basel) 13:171 33672250 10.3390/toxins13020171 PMC7927007

[5] Jia H, Liu N, Zhang Y, Wang C, Yang Y, Wu Z (2021) 3-Acetyldeoxynivalenol induces cell death through endoplasmic reticulum stress in mouse liver. Environ Pollut 286:117238 33984781 10.1016/j.envpol.2021.117238

[6] Ge L, Liu D, Mao X, Liu S, Guo J, Hou L, Chen X, Huang K (2022) Low dose of deoxynivalenol aggravates intestinal inflammation and barrier dysfunction induced by enterotoxigenic *Escherichia coli* infection through activating macroautophagy/NLRP3 inflammasomes. J Agric Food Chem 70:3009–3022 35201764 10.1021/acs.jafc.1c07834

[7] Vandenbroucke V, Croubels S, Verbrugghe E, Boyen F, De Backer P, Ducatelle R, Rychlik I, Haesebrouck F, Pasmans F (2009) The mycotoxin deoxynivalenol promotes uptake of *Salmonella Typhimurium* in porcine macrophages, associated with ERK1/2 induced cytoskeleton reorganization. Vet Res 40:64 19674540 10.1051/vetres/2009045

[8] Bimczok D, Doll S, Rau H, Goyarts T, Wundrack N, Naumann M, Danicke S, Rothkotter HJ (2007) The Fusarium toxin deoxynivalenol disrupts phenotype and function of monocyte-derived dendritic cells in vivo and in vitro. Immunobiology 212:655–666 17869643 10.1016/j.imbio.2007.05.002

[9] Gauthier T, Wache Y, Laffitte J, Taranu I, Saeedikouzehkonani N, Mori Y, Oswald IP (2013) Deoxynivalenol impairs the immune functions of neutrophils. Mol Nutr Food Res 57:1026–1036 23427020 10.1002/mnfr.201200755

[10] Liu D, Ge L, Wang Q, Su J, Chen X, Wang C, Huang K (2020) Low-level contamination of deoxynivalenol: a threat from environmental toxins to Porcine Epidemic Diarrhea Virus infection. Environ Int 143:105949 32673909 10.1016/j.envint.2020.105949 PMC7357974

[11] Pierron A, Vatzia E, Stadler M, Mair KH, Schmidt S, Stas MR, Durlinger S, Kreutzmann H, Knecht C, Balka G, Lagler J, Zaruba M, Rümenapf T, Saalmüller A, Mayer E, Ladinig A, Gerner W (2023) Influence of deoxynivalenol-contaminated feed on the immune response of pigs after PRRSV vaccination and infection. Arch Toxicol 97:1079–1089 36781434 10.1007/s00204-023-03449-9 PMC10025202

[12] Savard C, Pinilla V, Provost C, Gagnon CA, Chorfi Y (2014) In vivo effect of deoxynivalenol (DON) naturally contaminated feed on porcine reproductive and respiratory syndrome virus (PRRSV) infection. Vet Microbiol 174:419–426 25465662 10.1016/j.vetmic.2014.10.019

[13] Michiels A, Arsenakis I, Matthijs A, Boyen F, Haesaert G, Audenaert K, Eeckhout M, Croubels S, Haesebrouck F, Maes D (2018) Clinical impact of deoxynivalenol, 3-acetyl-deoxynivalenol and 15-acetyl-deoxynivalenol on the severity of an experimental *Mycoplasma hyopneumoniae* infection in pigs. BMC Vet Res 14:190 29914486 10.1186/s12917-018-1502-4 PMC6006720

[14] Gottschalk M, Segura M (2019) Streptococcocis. In: Zimmerman JJ, Karriker LA, Ramirez A, Schwartz KJ, Stevenson GW, Zhang J (eds) Diseases of swine, 11th edn. Wiley-Blackwell, Hoboken, NJ, USA, pp 934–950

[15] Gottschalk M, Xu J, Calzas C, Segura M (2010) *Streptococcus suis*: a new emerging or an old neglected zoonotic pathogen? Future Microbiol 5:371–391 20210549 10.2217/fmb.10.2

[16] Goyette-Desjardins G, Auger JP, Xu J, Segura M, Gottschalk M (2017) *Streptococcus suis*, an important pig pathogen and emerging zoonotic agent-an update on the worldwide distribution based on serotyping and sequence typing. Emerg Microbes Infect 3:e45 10.1038/emi.2014.45 PMC4078792 26038745

[17] Segura M, Fittipaldi N, Calzas C, Gottschalk M (2017) Critical *Streptococcus suis* virulence factors: are they all really critical? Trends Microbiol 25:585–599 28274524 10.1016/j.tim.2017.02.005

[18] Xia X, Qin W, Zhu H, Wang X, Jiang J, Hu J (2019) How *Streptococcus suis* serotype 2 attempts to avoid attack by host immune defenses. J Microbiol Immunol Infect 52:516–525 30954397 10.1016/j.jmii.2019.03.003

[19] Bleuze M, Gottschalk M, Segura M (2021) Neutrophils in *Streptococcus suis* infection: from host defense to pathology. Microorganisms 9:2392 34835517 10.3390/microorganisms9112392 PMC8624082

[20] Obradovic MR, Segura M, Segales J, Gottschalk M (2021) Review of the speculative role of co-infections in *Streptococcus suis*-associated diseases in pigs. Vet Res 52:49 33743838 10.1186/s13567-021-00918-w PMC7980725

[21] Guan X, Martinez AR, Fernandez M, Molist F, Wells JM (2024) Santos RR (2024) The mycotoxins T-2 and deoxynivalenol facilitate the translocation of* Streptococcus sui*s across porcine Ileal organoid monolayers. Toxins 16:382 39330840 10.3390/toxins16090382 PMC11436090

[22] Mutagenesis of *Streptococcus equis* and *Streptococcus suis* by transposon Tn917. 12695044 10.1016/s0378-1135(03)00030-0

[23] Lecours MP, Gottschalk M, Houde M, Lemire P, Fittipaldi N, Segura M (2011) Critical role for *Streptococcus suis* cell wall modifications and suilysin in resistance to complement-dependent killing by dendritic cells. J Infect Dis 204:919–929 21849289 10.1093/infdis/jir415

[24] Obradovic M, Corseaut L, Dolbec D, Gottschalk M, Segura M (2021) Experimental evaluation of protection and immunogenicity of *Streptococcus suis* bacterin-based vaccines formulated with different commercial adjuvants in weaned piglets. Vet Res 52:133 34666827 10.1186/s13567-021-01004-x PMC8527783

[25] Lecours MP, Segura M, Lachance C, Mussa T, Surprenant C, Montoya M, Gottschalk M (2011) Characterization of porcine dendritic cell response to *Streptococcus suis*. Vet Res 42:72 21635729 10.1186/1297-9716-42-72 PMC3127767

[26] Martelet L, Lacouture S, Goyette-Desjardins G, Beauchamp G, Surprenant C, Gottschalk M, Segura M (2017) Porcine dendritic cells as an in vitro model to assess the immunological behaviour of *Streptococcus suis* subunit vaccine formulations and the polarizing efect of adjuvants. Pathogens 6:13 28327531 10.3390/pathogens6010013 PMC5371901

[27] Pageaut H, Lacouture S, Lehoux M, Marois-Crehan C, Segura M, Gottschalk M (2023) Interactions of *Mycoplasma hyopneumoniae* and/or *Mycoplasma hyorhinis* with *Streptococcus suis* serotype 2 using in vitro co-infection models with swine cells. Pathogens 12:866 37513713 10.3390/pathogens12070866 PMC10383509

[28] Corsaut L, Misener M, Canning P, Beauchamp G, Gottschalk M, Segura M (2020) Field study on the immunological response and protective effect of a licensed autogenous vaccine to control *Streptococcus suis* infections in post-weaned piglets. Vaccines 8:384 32674276 10.3390/vaccines8030384 PMC7565864

[29] Mathieu-Denoncourt A, Letendre C, Auger JP, Segura M, Aragon V, Lacouture S, Gottschalk M (2018) Limited interactions between *Streptococcus suis* and *Haemophilus parasuis* in in vitro co-infection studies. Pathogens 7:7 29316613 10.3390/pathogens7010007 PMC5874733

[30] National Swine Nutrition Guide, Tables on nutrient recommendations, ingredient composition, and use rates. Pork Information Gateway (2010) [Available from: https://porkgateway.org/resource/national-swine-nutrition-guide-tables-on-nutrient-recommendations-ingredient-composition-and-use-rates/.

[31] Valenta H, Danicke S, Doll S (2003) Analysis of deoxynivalenol and de-epoxy-deoxynivalenol in animal tissues by liquid chromatography after clean-up with an immunoaffinity column. Mycotoxin Res 19:51–55 23604669 10.1007/BF02940093

[32] Segura MA, Cleroux P, Gottschalk M (1998) *Streptococcus suis* and group B *Streptococcus* differ in their interactions with murine macrophages. FEMS Immunol Med Microbiol 21:189–195 9718208 10.1111/j.1574-695X.1998.tb01165.x

[33] Vanhoutte I, De Mets L, De Boevre M, Uka V, Di Mavungu JD, De Saeger S, De Saeger S, De Gelder J, Audenaert K (2017) Microbial detoxification of Deoxynivalenol (DON), assessed via a *Lemna minor* L. bioassay, through biotransformation to 3-epi-DON and 3-epi-DOM-1. Toxins 9:63 28208799 10.3390/toxins9020063 PMC5331442

[34] Dame-Korevaar A, Gielen C, van Hout J, Bouwknegt M, Faba L, Vrieling M (2025) Quantification of antibiotic usage against *Streptococcus suis* in weaner pigs in the Netherlands between 2017 and 2021. Prev Vet Med 235:106400 39644828 10.1016/j.prevetmed.2024.106400

[35] Estrada AA, Gottschalk M, Rendahl A, Rossow S, Marshall-Lund L, Marthaler DG, Gebhart CJ (2021) Proposed virulence-associated genes of *Streptococcus suis* isolates from the United States serve as predictors of pathogenicity. Porcine Health Manag 7:22 33648592 10.1186/s40813-021-00201-6 PMC7917538

[36] Kim SW, Holanda DM, Gao X, Park I, Yiannikouris A (2019) Efficacy of a yeast cell wall extract to mitigate the effect of naturally co-occurring mycotoxins contaminating feed ingredients fed to young pigs: impact on gut health, microbiome, and growth. Toxins Basel 11:633 31683617 10.3390/toxins11110633 PMC6891535

[37] Liu D, Wang Q, He W, Ge L, Huang K (2022) Deoxynivalenol aggravates the immunosuppression in piglets and PAMs under the condition of PEDV infection through inhibiting TLR4/NLRP3 signaling pathway. Ecotoxicol Environ Saf 231:113209 35051765 10.1016/j.ecoenv.2022.113209

[38] Sun Y, Song Y, Long M, Yang S (2023) Immunotoxicity of three environmental mycotoxins and their risks of increasing pathogen infections. Toxins 2023(15):187 10.3390/toxins15030187 PMC10054902 36977078

[39] Liu D, Wang Q, He W, Chen X, Wei Z, Huang K (2020) Two-way immune effects of deoxynivalenol in weaned piglets and porcine alveolar macrophages: due mainly to its exposure dosage. Chemosphere 249:126464 32229367 10.1016/j.chemosphere.2020.126464

[40] Maier T, Guell M, Serrano L (2009) Correlation of mRNA and protein in complex biological samples. FEBS Lett 583:3966–3973 19850042 10.1016/j.febslet.2009.10.036

[41] Nossol C, Landgraf P, Oster M, Kahlert S, Barta-Boszormenyi A, Kluess J, Wimmers K, Isermann B, Stork O, Dieterich DC, Dänicke S, Rothkötter HJ (2024) Deoxynivalenol triggers the expression of IL-8-related signaling cascades and decreases protein biosynthesis in primary monocyte-derived cells. Mycotoxin Res 40:279–293 38498144 10.1007/s12550-024-00528-3 PMC11043135

[42] Shandilya UK, Sharma A, Xu R, Muniz MMM, Karrow NA (2023) Evaluation of immunomodulatory effects of *Fusarium* mycotoxins using bacterial endotoxin-stimulated bovine epithelial cells and macrophages in co-culture. Genes Basel 14:2024 38002956 10.3390/genes14112014 PMC10671659

[43] Gu C, Gao X, Guo D, Wang J, Wu Q, Nepovimova E, Wu W, Kuca K (2021) Combined effect of Deoxynivalenol (DON) and *Porcine circovirus type 2* (Pcv2) on inflammatory cytokine mRNA expression. Toxins 13:422 34199278 10.3390/toxins13060422 PMC8231776

[44] Savard C, Provost C, Alvarez F, Pinilla V, Music N, Jacques M, Gagnon CA, Chorfi Y (2015) Effect of deoxynivalenol (DON) mycotoxin on in vivo and in vitro porcine circovirus type 2 infections. Vet Microbiol 176:257–267 25717015 10.1016/j.vetmic.2015.02.004

[45] Auger JP, Fittipaldi N, Benoit-Biancamano MO, Segura M, Gottschalk M (2016) Virulence studies of different sequence types and geographical origins of *Streptococcus suis* serotype 2 in a mouse model of infection. Pathogens 5:48 27409640 10.3390/pathogens5030048 PMC5039428

[46] Vandenbroucke V, Croubels S, Martel A, Verbrugghe E, Goossens J, Van Deun K, Boyen F, Thompson A, Shearer N, De Backer P, Haesebrouck F, Pasmans F (2011) The mycotoxin deoxynivalenol potentiates intestinal inflammation by *Salmonella typhimurium* in porcine ileal loops. PLoS One 6:e23871 21909370 10.1371/journal.pone.0023871 PMC3166085

[47] Jin W, Park SY, Kim YH, Kim SJ, Han JH (2024) Occurrence of mycotoxins in swine feed from South Korea. J Adv Vet Anim Res 11:125–131 38680795 10.5455/javar.2024.k756 PMC11055581

[48] Almeida I, Martins HM, Santos S, Costa JM, Bernardo F (2011) Co-occurrence of mycotoxins in swine feed produced in Portugal. Mycotoxin Res 27:177–181 23605797 10.1007/s12550-011-0093-8

