# Supplementary material for: Lack of significant impact of deoxynivalenol (DON) on both swine dendritic cell activities and systemic infection caused by a virulent Streptococcus suis serotype 2 strain
Source: Vet Res. 2026 Apr 7;57:69. doi: 10.1186/s13567-026-01745-7 (PMC13154709; doi:10.1186/s13567-026-01745-7)
Supplement: Supplementary file 1 — Additional file 1 Effect of DON-contaminated feed on blood bacterialburden afterS. suisinfection. [file 13567_2026_1745_MOESM1_ESM.pptx]

## Slide 1
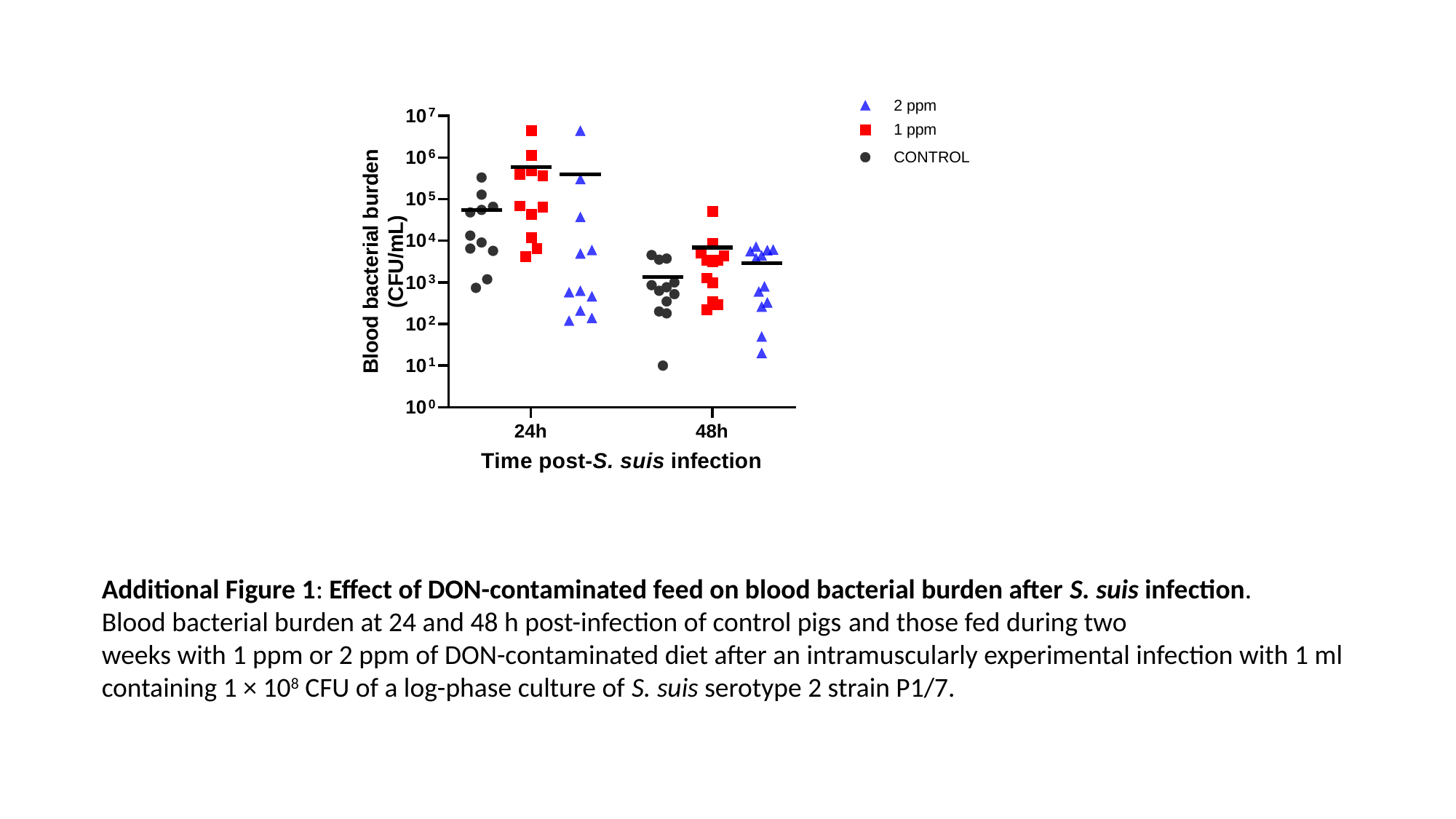

Additional Figure 1: Effect of DON-contaminated feed on blood bacterial burden after S. suis infection.
Blood bacterial burden at 24 and 48 h post-infection of control pigs and those fed during two
weeks with 1 ppm or 2 ppm of DON-contaminated diet after an intramuscularly experimental infection with 1 ml
containing 1 × 10^8^ CFU of a log-phase culture of S. suis serotype 2 strain P1/7.
